# Psychometric properties of the Norwegian version of the clinical learning environment comparison survey

**DOI:** 10.1002/nop2.742

**Published:** 2020-12-23

**Authors:** Camilla Olaussen, Lars‐Petter Jelsness‐Jørgensen, Christine Raaen Tvedt, Dag Hofoss, Ingunn Aase, Simen A. Steindal

**Affiliations:** ^1^ Lovisenberg Diaconal University College Oslo Norway; ^2^ The University of Stavanger Stavanger Norway; ^3^ Department of Health and Social Studies Østfold University College Fredrikstad Norway; ^4^ Østfold Hospital Trust Kalnes Norway

**Keywords:** clinical education, learning needs, nursing education, psychometrics, simulation, translation

## Abstract

**Aim:**

To translate The Clinical Learning Environment Comparison Survey (CLECS) into Norwegian and to evaluate the psychometric properties of the Norwegian version.

**Design:**

A cross‐sectional survey including a longitudinal component.

**Methods:**

The CLECS was translated into Norwegian following the World Health Organization guidelines, including forward translation, expert panel, back‐translation, pre‐testing and cognitive interviewing. Nursing students at a Norwegian university college were invited to participate in the study (psychometrical testing) based on informed consent. Reliability and validity of the translated version of CLECS were investigated using a confirmatory factor analysis (CFA), Cronbach's alpha and test–retest analysis.

**Results:**

A total of 122 nursing students completed the questionnaire and Cronbach alphas for the CLECS subscales ranged from 0.69 to 0.89. CFA goodness‐of‐fit indices (χ^2^/*df* = 1.409, CFI = 0.915, RMSEA = 0.058) showed acceptable model fit. Test–retest ICC ranged from 0.55 to 0.75, except for two subscales with values below 0.5

## 1 INTRODUCTION

It is well known that nursing students may experience challenges related to learning in clinical practice placements, such as lack of qualified supervision, limited clinical time and limited access to adequate learning experiences (Arkan et al., 2018; Morrell & Ridgway, 2014; Richardson et al., 2014). To ensure an adequate clinical nursing education, the use of patient simulation as a learning strategy has increased considerably worldwide (Breymier et al., 2015; Gates et al., 2012; Hayden et al., 2014). Patient simulation may ensure that nursing students receive high‐quality and complex learning situations, something that cannot be guaranteed in the traditional clinical practice placements (Gates et al., 2012). Moreover, systematic reviews have found that simulation training may improve students’ knowledge levels, clinical skills and general nursing competences (Cant & Cooper, 2017; Haddeland et al., 2018) and some researchers have also recommended simulation as a substitute for clinical hours among nursing students (Gates et al., 2012; Hayden et al., 2014; Soccio, 2017). To evaluate the clinical and simulated practice so that both strategies can be optimally combined in nursing education programmes, valid evaluation tools are needed (Gu et al., 2018).

### 1.1 Background

The Clinical Learning Environment Comparison Survey (CLECS) was primarily developed to provide empirical data to guide the use of patient simulation in nursing education as an alternative to clinical practice for nursing students (Leighton, 2015).

According to Leighton (2015), the CLECS may be a valuable instrument for course evaluation, programme evaluation and assessment of student learning in nursing education. The instrument addresses how students perceive that learning needs are met in the clinical versus the simulated environment by rating each environment side by side on 27 items related to clinical learning. In CLECS, students are asked about their experiences with communication, the nursing process, sense of holism, critical thinking, self‐efficacy and teaching–learning dyad. Data about such issues are important for nursing educators to evaluate whether teaching strategies both in the clinical and in the simulated environment are effective. The CLECS has previously been used in a national randomized controlled study in the US, where 666 nursing students completed the instrument at the end of each clinical course and again at the end of the programme to rate how well each environment met the students’ learning needs (Hayden et al., 2014). The identification of unmet learning needs of students should drive changes in how nursing educators manage those learning environments, thereby having an impact on the learning outcomes (Leighton, 2015).

To be used in another language and culture, the instrument needs to undergo translation and psychometrical evaluation. One previous Chinese study has assessed the psychometric properties of the CLECS in another language and context than the original. The CLECS (Chinese version) showed satisfactory reliability and validity among Chinese undergraduate nursing students (Gu et al., 2018). Equivalence between the original and translated versions is crucial to ensure that conclusions drawn from the use of a translated instrument are based on differences and similarities between cultures on the phenomenon being measured and not on errors in translation (Wang et al., 2006). Hence, systematic and precise translation and contextual evaluation are required to ensure internationally comparable results (Gudmundsson, 2009).

The present study was driven by the increasing use of patient simulation in Norwegian nursing education and the need for valid tools to guide educators in their work to develop simulation experiences that may compensate for learning needs that are not properly met in the clinical environment. Therefore, the aim of this study was to translate the Clinical Learning Environment Comparison Survey (CLECS) into Norwegian and to evaluate its psychometric properties.

## 2 METHODS

### 2.1 Design

The research design is a cross‐sectional survey including a longitudinal component.

### 2.2 CLECS

CLECS was developed and validated by Leighton (2015) and consists of 27 items, distributed on six subscales as presented in Table 1. For each of the items, level of agreement is scored using a four‐point Likert scale: 1 = “Not met,” 2 = “Partially met,” 3 = “Met”; to 4 = “Well met,” in addition to the alternative “Not applicable.” For each item, the students set a score for both the traditional clinical environment and the simulated environment and allow evaluation of each environment score separately and comparison to be made between the environment scores (Leighton, 2015).

**TABLE 1:** The hypothesized factor model and corresponding items in CLECS (Leighton, 2015)

Survey subscales	Survey items
Communication (4 items)	1. Preparing to care for patient 2. Communicating with interdisciplinary team 3. Interacting with patient 4. Providing information and support to patient's family
Nursing Process (6 items)	5. Understanding rationale for patient's treatment plan 6. Understanding patient's pathophysiology 7. Identifying patient's problems 8. Implementing care plan 9. Prioritizing care 10. Performing appropriate assessment
Holism (6 items)	11. Assessing outcomes of the care provided 12. Identifying short‐ and long‐term nursing goals 13. Discussing patient's psychosocial needs 14. Discussing patient's developmental needs 15. Discussing patient's spiritual needs 16. Discussing patient's cultural needs
Critical Thinking (2 items)	17. Anticipating and recognizing changes in patient's condition 18. Taking appropriate action when patient's condition changes
Self‐Efficacy (4 items)	19. Reacting calmly to changes in my patient's condition 20. Knowing what to do if I make an error in my care 21. Being confident in my decisions 22. Feeling confident in my nursing abilities
Teaching–Learning Dyad (5 items)	23. Having my instructor available to me 24. Feeling challenged and stimulated 25. Receiving immediate feedback on performance 26. Feeling supported by instructor and peers when making care related decisions 27. Improving my critical thinking skills with experience

There is no established method to score the CLECS. In the present study, respondent subscale scores were calculated by summing the respondent's answers to the items included in the subscale and dividing that sum by his/her number of answers on the subscale. Higher scores indicate that learning needs are met, and lower scores indicate that learning needs are not met.

### 2.3 Translation procedure

Permission to translate, validate and use the CLECS was obtained from the developer by email communication with the first author (CO). The translation process followed the guidelines from The World Health Organization (WHO, 2018), including forward translation, use of an expert panel, back‐translation, pre‐testing and cognitive interviewing.

#### 2.3.1 Forward translation

The forward translation was made independently by two translators, both registered nurses and nursing teachers, familiar with the terminology of the area covered by the CLECS (Wild et al., 2005). The translators’ mother tongue was Norwegian, and both were fluent in English.

#### 2.3.2 Expert panel

An expert panel was established to identify and resolve inadequate expressions and concepts of the translations between the original version of the CLECS and the forward translations (WHO, 2018). The panel consisted of five members: the two original translators and three experienced nursing teachers, all registered nurses, familiar with the terminology of the area covered by the CLECS. Two were experienced within instrument development and instrument translation. In case of disagreement between the two translated versions, the expert panel resolved the discrepancies seeking agreement and reconciled the translations into a single forward translation.

#### 2.3.3 Back‐translation

The back‐translation was made by a professional translator and native speaker of English. The back‐translator had no former knowledge of the CLECS and did not see the source version before or during the back‐translation (Wild et al., 2005). Following back‐translation, the translated version was sent to the developer together with the original CLECS. In one item (item 14: Discussing the patients developmental needs), the two versions differed in conceptual meaning. Based on feedback from the developer, the wording in item 14 was subsequently changed by the expert panel.

#### 2.3.4 Pre‐testing and cognitive interviewing

Nine nursing students in the second year of their bachelor education were invited by the first author (CO) to pre‐test the Norwegian version of the CLECS in an email. Six students accepted the invitation, pre‐tested the instrument and attended a focus group interview. Beforehand, the students had been exposed to both clinical practice and simulation training environments in their educational programme of nursing. The pre‐test of the CLECS took approximately 15 min to complete.

An experienced interviewer (SAS) conducted the interview, while the first author (CO) took notes. The students were asked to evaluate the structure of the CLECS (Norwegian version) such as the order of questions and response options, layout and length. They were asked to evaluate the meaning of the questions, the wording and whether the directions for completing the test was clear (Willis, 2005). For each item, students were asked what they thought the items were asking for, how they would rephrase the items in their own words and what came to their mind when they heard a particular phrase or term. Finally, when alternative expressions existed for an item, the students were asked to choose which alternative conformed better to their usual language. The students found the translated CLECS easy to understand and did not consider alternative expressions better than those suggested.

### 2.4 Psychometric testing of the CLECS (Norwegian version)

Data for the psychometric testing of the CLECS (Norwegian version) were collected during the spring and fall of 2019 at a university college in Norway that provide bachelor education in nursing. A convenience sampling method was used. The study population was all first‐year nursing students (139) in their second semester of the education programme that had at least one patient simulation experience as suggested in the original CLECS (Leighton, 2015). The students had also finished a 7‐week mandatory clinical practice period in nursing homes. The students were informed orally and by email about the study beforehand. The volunteers signed a written informed consent before answering a paper version of the CLECS distributed at the university college's simulation centre.

The retest of the CLECS was distributed electronically after 14 days to respondents who had accepted to participate in the retest. The time span of 14 days was chosen to avoid conflicts with other student activities/assignments.

### 2.5 Ethical considerations

The Norwegian Social Science Data Services approved the study (ref. number 956321). Participation to the test and retest was based on written informed consent and performed in accordance with the 2013 revised version of the Declaration of Helsinki.

### 2.6 Statistical analyses

#### 2.6.1 Confirmatory factor analysis: internal construct validity

The CLECS developer specified a six‐factor model, as presented in Table 1 (Leighton, 2015). We performed a confirmatory factor analysis (CFA) to investigate whether the pre‐hypothesized factor model fitted our observed data. The fit of the hypothesized model was assessed by these goodness‐of‐fit indices: the chi‐square/*df* ratio, the p, the Comparative Fit Index (CFI), the root mean square error of approximation (RMSEA) and the *p*_close_.

Acceptable goodness‐of‐fit values indicate internal construct validity of the model. Carmines and Mclver (1981) consider chi‐square/*df* ratios of 2–3 as acceptable, whereas Byrne (1989) will not accept ratios above 2. The *p*‐values should exceed .05 (Browne & Cudeck, 1993). CFI values range from 0 to 1 and should be at least 0.90: larger values indicate better fit (Bryant & Yarnold, 1995). A root mean square error of approximation (RMSEA) not exceeding 0.08 indicates adequate model fit and below 0.05 close fit (Browne & Cudeck, 1993). As the RMSEA value is a sample‐based estimate, larger RMSEA values may hide an acceptable model fit. A non‐significant *p*_close_ value says that the RMSEA does not exceed the 0.05 RMSEA limit, which indicates acceptable model fit—and a *p*_close_ value of above .10 indicates good fit (Schermelleh‐Engel & Moosbrugger, 2003).

#### 2.6.2 Internal consistency

Internal consistency was assessed by Cronbach alphas, and values exceeding 0.7 were classified as good (Streiner, 2003). We also determined whether all items were contributing to the scales they were assumed to belong to by computing the Cronbach alpha value if the item was deleted. Additionally, we checked that all items were more highly correlated with the factor they were assumed to belong to (CITC) than with any other factor.

#### 2.6.3 Test–retest reliability

Test–retest reliability was assessed by the intraclass correlation coefficient (ICC). ICC estimates with 95% confident intervals were calculated based on a single rater measurement, absolute‐agreement, 2‐way mixed‐effects model. The ICC varies from 0 to 1, where 1 is equivalent to perfect reliability. ICC values less than 0.5 are indicative of poor test–retest reliability, values between 0.5 and 0.75 indicate moderate reliability, values between 0.75 and 0.9 indicate good reliability, and values greater than 0.90 indicate excellent reliability (Koo & Li, 2016).

While CFA was performed using AMOS Graphics (an IBM SPSS module), all other analyses were performed using IBM SPSS Statistics version 26 (IBM). The psychometric analyses were based on data from the clinical environment.

## 3 RESULTS

Of 139 students invited to participate, 122 (87.7%) returned the instrument at the baseline. The mean age of the 122 respondents at baseline was 23.6 years (*SD* 4.8), and 102 (83.6%) were female. Of the 89 students who had agreed to participate in the retest, 40 (45%) returned the instrument at follow‐up. The mean age of the 40 retest respondents was 23.9 years (*SD* 5.1), and 32 (80%) were female. The response interval for the retest ranged from 2 to 8 weeks.

Completing the instrument at the baseline took 10 to 15 min. Answers were moderately skewed towards the “fully agree” end of the scale, but a full range of responses was observed. Missing at item level (not including the “Not applicable” alternative) was on average 1.4% (range of 0 to 4.1%), counting both the clinical and the simulated environment. A high frequency of “Not applicable” answers from the simulated environment made the data from the simulated environment insufficient for psychometric testing. For the simulated environment data, the “Not applicable” alternative was chosen 235 times, while for the practice environment data it was chosen 26 times.

### 3.1 Internal consistency

Cronbach alphas for each clinical environment subscale are presented in Table 2. The exclusion of any item from its own subscale would not noticeably increase the α‐values. Almost all items in the clinical environment (97%) were more strongly correlated with the subscale under which the hypothesized CFA model subsumed them. The exceptions were item 1: Preparing to care for patient, which was more strongly correlated with the Nursing Process, item 11: Assessing outcomes of the care provided, which was more strongly correlated with the Nursing process and Critical thinking and item 12: Identifying short‐ and long‐term nursing goals, which was more strongly correlated with the Nursing process.

**TABLE 2:** Mean score and Cronbach's alpha by subscale (*N* = 122)

Subscale/factor	Mean score (*N*)	*SD*	Min–Max	Cronbach´s Alpha
Communication	3.21 (121)	0.54	1.25–4.00	0.69
Nursing Process	3.09 (121)	0.67	1.17–4.00	0.89
Holism	2.72 (121)	0.64	1.00–4.00	0.81
Critical Thinking	3.23 (122)	0.68	1.00–4.00	0.76
Self‐Efficacy	2.95 (122)	0.64	1.50–4.00	0.83
Teaching–Learning Dyad	3.03 (121)	0.68	1.40–4.00	0.83

Abbreviation: *SD*, Standard deviation.

### 3.2 Test–retest reliability

For four of the six subscales in the clinical environment test–retest, ICC exceeded 0.5 as shown in Table 3. For two of the subscales, Communication and Critical Thinking, test–retest ICC were below 0.5. Test–retest ICC calculated at item level in Communication for item 1: Preparing to care for patient and item 2: Communicating with interdisciplinary team, were especially low, respectively 0.38 and 0.35. When item 1, or item 2, was removed from the subscale, the ICC estimate increased to 0.67. For Critical Thinking, two items (item 17: Anticipating and recognizing changes in patient's condition and item 18: Taking appropriate action when patient's condition changes) were low 0.37 and 0.40, respectively.

**TABLE 3:** Test–retest of the CLECS (Norwegian version) in patients with complete data sets at both times of measurement

Test–retest, intraclass correlation coefficient by subscale (*N* = 40)						
Subscales	Baseline Mean (*SD*)	Retest Mean (*SD*)	Mean difference (95% CI)	ICC	95% CI	
					Lower	Upper
Communication	3.28 (0.48)	3.10 (0.60)	0.18 (−0.01–0.37)	0.41	0.12	0.63
Nursing Process	3.05 (0.68)	3.00 (0.64)	0.05 (−12–0.22)	0.68	0.47	0.82
Holism	2.78 (0.62)	2.86 (0.66)	−0.82 (−0.23–0.07)	0.72	0.54	0.84
Critical Thinking	3.36 (0.66)	3.21 (0.67)	0.15 (−0.09–0.40)	0.42	0.11	0.65
Self‐Efficacy	3.06 (0.73)	2.93 (0.66)	0.13 (−0.09–0.35)	0.55	0.28	0.74
Teaching–Learning Dyad	3.04 (0.71)	3.01 (0.85)	0.03 (−0.15–0.22)	0.75	0.57	0.93

Abbreviations: CI, confidence interval; ICC, intraclass correlation coefficient. Calculated on a single rater measurement, absolute‐agreement, 2‐way mixed‐effects model.

### 3.3 Construct validity: goodness‐of‐fit values for the confirmatory factor analysis model

**FIGURE 1:**
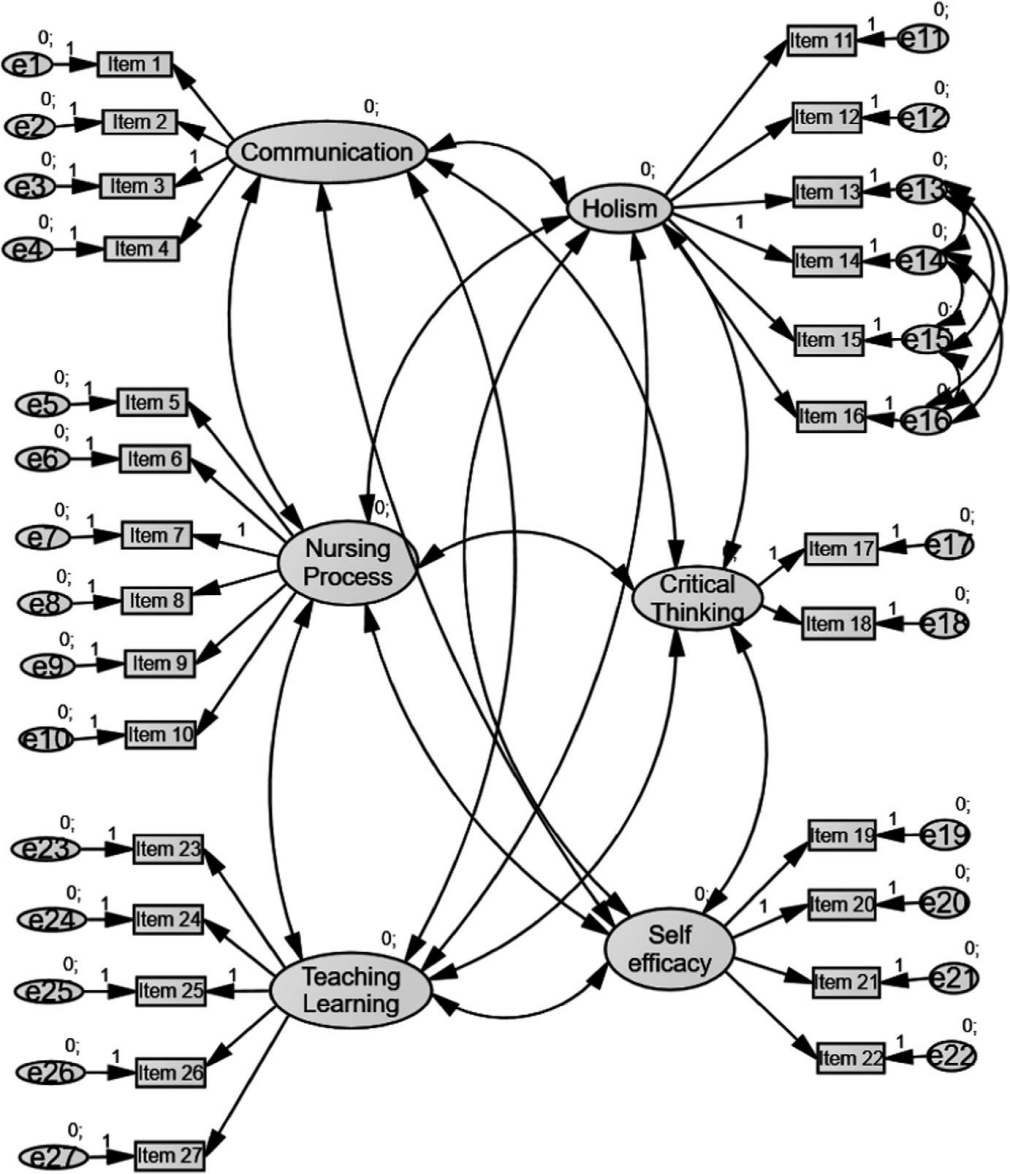
Factor structure model for the CLECS (Norwegian version) (*N* = 122)

The factor structure model of the CLECS (Norwegian version) is presented in Figure 1. The content of the items is presented in Table 1. The model was developed with a randomly selected 50% of the data set from the clinical environment and was confirmed by being tested on the entire clinical environment data set (Pohlmann, 2004).

Goodness‐of‐fit indices for the factor structure model are shown in Table 4.

**TABLE 4:** Goodness‐of‐fit indices (*N* = 122)

χ^2^	Df	χ^2^/*df*	*p*	CFI	RMSEA	*p*_close_
427.03	303	1.409	<.001	0.915	0.058	.150

Abbreviations: CFI, the Comparative Fit Index; *df*, degrees of freedom; *p*, *p* value; RMSEA, the root mean square error of approximation; χ^2^, the chi‐square; χ^2^/*df*, the chi‐square to *df* ratio.

## 4 DISCUSSION

The Norwegian version of CLECS showed good acceptability, acceptable construct validity and a good internal consistency. While most subscales displayed moderate to good test–retest reliability, problematic reliability was observed in the subscales Communication and Critical thinking.

In the original CLECS, the lowest Cronbach alpha score for all the subscales was 0.73 (Leighton, 2015). In the present study, the relatively high Cronbach alphas for all hypothesized subscales, except for the Communication subscale, demonstrated internal consistency. The exclusion of any item from its own subscale did not significantly increase the α‐value. Moreover, the fact that almost all items were more strongly correlated with their own subscale than with any of the other subscales confirms that responses were grouped in the way hypothesized by our model.

Leighton (2015) evaluated test–retest reliability in the original CLECS by Pearson's correlation coefficient r, while in the current study, test–retest reliability was assessed by ICC. The advantage of the latter approach is that ICC will not only discover within‐subject change in scores but also a possible collective change in scores among respondents in a group over time. While Leighton (2015) only found two subscales (Holism and Teaching–learning dyad) in the original CLECS with values above 0.5 indicating a moderate test–retest reliability, three subscales in the present study indicated a moderate reliability (Nursing Process, Holism and Self‐Efficacy) and one (Teaching–Learning dyad) indicated good test–retest reliability (Koo & Li, 2016).

Two subscales (Communication and Critical Thinking) had values indicating poor test–retest reliability (<0.5). The low correlations in these subscales were caused by four items (item 1: Preparing to care for patient and 2: Communicating with interdisciplinary team in Communication, item 17: Anticipating and recognizing changes in patient's condition and 18: Taking appropriate action when patient's condition changes in Critical Thinking). Low test–retest correlations may be caused by instrument instability such as problematic words, topics or expressions (Blacker & Endicott, 2008; Furr & Bacharach, 2008). As Norwegian nursing education is heavily influenced by US textbooks and research articles, it was not difficult to find Norwegian words and expressions that captured the CLECS original meaning in the translation process. The group of second‐year students that evaluated the CLECS (Norwegian version) confirmed the importance and relevance of the topics and wording, suggesting that the CLECS may be used in a Norwegian context. However, it may have been easier to detect potential problems in the translated version using individual interviews instead of a focus group interview (Gjersing et al., 2010). Furthermore, the differences in responses may be due to the sample´s characteristics (Blacker & Endicott, 2008; Furr & Bacharach, 2008). The second‐year students may have been more familiar with nursing terminology and nursing care than the first‐year respondents used in the psychometric testing. The low test–retest correlations may have reflected an uncertainty on how to interpret and evaluate some of the CLECS’s rather complex topics due to an early stage in the nurse education programme.

Test–retest reliability estimates will also depend on test–retest intervals, test conditions and true change in the variables of interest (Blacker & Endicott, 2008; Furr & Bacharach, 2008). The time interval between the tests must be short enough to ensure a minimal, or no change in the individual, but long enough to avoid the risk of recall bias (Blacker & Endicott, 2008). Several retest results in this study were returned after longer retest intervals and time may have bleached these respondents’ recollection and attenuated their evaluation of their experiences. An assumption in test–retest reliability is also that the error variance of the first measurement is equal to the error variance in the second measurement—which requires identical test conditions. We were not able to create two identical testing situations, and thus, we could not control for extraneous variables such as noise or distractions, which can affect responses in random ways and mask the differences among the respondents’ true scores (Furr & Bacharach, 2008). The unequal test conditions made it impossible to determine whether differences in scores from test to retest were due to “true” differences or to “chance” errors. In future studies of CLECS (Norwegian version), the test–retest reliability in the subscales Communication and Critical Thinking should be further evaluated.

The construct validity of the CLECS (Norwegian version), as judged by the goodness‐of‐fit indicators from CFA, can be considered acceptable. In the original CLECS, CFA was used to test and revise subscale compositions (Leighton, 2015), resulting in the hypothesized factor structure model used in this study. We did not re‐define the hypothesized model. However, one minor adjustment was done; we linked the error terms of items 13 to 16 as these questions all contained the possibly ambiguous word “diskutere” (discuss). In each item, the word “discuss” may be have been read as “myself thinking it through in my head” or as “I talked it over with some other person.” As all four items carry the same interpretational uncertainty, their error terms may be related. One goodness‐of‐fit indicator that speaks against the fit of the model is the χ^2^’s *p*‐value of less than .001. However, the χ^2^‐*df* ratio was well below the limit recommended by Byrne (1989). The *p*_close_ and the RMSEA both met the criteria suggested by Browne and Cudeck (1993).

The results of this study show that the CLECS has potential as an instrument for assessment of student learning in the Norwegian nursing education. An important step in improving nursing students’ clinical education is to understand how learning needs are met by the two methods of learning. The CLECS could be integrated in Norwegian nursing education for course and programme evaluations. CLECS findings may also be used to guide nursing educators in their work to develop and refine simulation experiences that may compensate for students learning challenges in clinical practice (Gu et al., 2018; Leighton, 2015).

Until now, no valid instrument that provides educators the direction on how to ensure an optimal combination of clinical and simulated experiences has been available in Norway. In this first Norwegian translation and testing of the CLECS, the internal reliability and goodness‐of‐fit results are based on observed data from the clinical environment. We were unable to evaluate reliability and the factor structure model for the simulation environment because many respondents lacked sufficient simulation experience and therefore too often ticked the “Not applicable” alternative in subscale items that did not match the content in their simulation experiences. Although the suggested minimum number of simulation experiences for the CLECS is set to one, the instrument subscales may be more suited for respondents with a broader simulation experience.

The internal consistency and construct validity tests were performed on a relatively small sample of 122 respondents. There is near universal agreement that factor analyses are inappropriate when sample sizes are below 50 (Garson, 2013). The suggested minimum size for conducting factor analysis differ in absolute numbers from 100 to over 1,000 and, in relative terms, from 3 to 20 times the number of variables (Mundfrom et al., 2005). Bryant and Yarnold (1995) suggest that the subjects‐to‐variables ratio should be no lower than 5. This criterion would have required 135 respondents, which was aimed for, but not quite reached, in this study: our subjects‐to‐variables ratio was 4.5:1. The original CLECS has no established scoring method, leaving the decision on how to score the instrument to the user, which may make it difficult to compare CLECS results nationally and internationally.

## 5 CONCLUSION

The CLECS (Norwegian version) has potential as a useful instrument to measure nursing students’ perceptions of how well their learning needs are met. The hypothesized six‐factor model had acceptable construct validity, good internal consistency and most subscales displayed moderate to good test–retest reliability. However, low test–retest values in two of the subscales revealed a need to further investigate these aspects. Also, future research should confirm the factor structure on data from the simulated environment—and preferably with data collected from respondents with a broader simulation experience.

## CONFLICT OF INTEREST

None.

## AUTHOR CONTRIBUTIONS

CO was responsible for the conception, design, analysis, and evaluation of the results and worked out the drafts and completed the submitted version of the manuscript. LPJ, CRT, DH IA and SAS contributed to conception and design in addition to participate in the analyses and evaluation of the results and contributed to the manuscripts intellectual content and critical review. All authors have given their final approval for the submitted version.

## ETHICS APPROVAL AND CONSENT TO PARTICIPATE

The Norwegian Social Science Data Services approved the study (ref. number 956321). Participation to the test and retest was based on written informed consent.

## Data Availability

Data not available due to ethical restrictions.
